# Retained avidity despite reduced cross-binding and cross-neutralizing antibody levels to Omicron after SARS-COV-2 wild-type infection or mRNA double vaccination

**DOI:** 10.3389/fimmu.2023.1196988

**Published:** 2023-07-21

**Authors:** Teresa Harthaller, Barbara Falkensammer, David Bante, Maria Huber, Melanie Schmitt, Habib Benainouna, Annika Rössler, Verena Fleischer, Dorothee von Laer, Janine Kimpel, Reinhard Würzner, Wegene Borena

**Affiliations:** ^1^ Department of Hygiene, Microbiology and Public Health, Institute of Virology, Innsbruck Medical University, Innsbruck, Austria; ^2^ Department of Hygiene, Microbiology and Public Health, Institute of Hygiene and Medical Microbiology, Innsbruck Medical University, Innsbruck, Austria

**Keywords:** COVID-19, binding antibodies, avidity, mutation, humoral immunity, convalescent, S1 domain, ancestral strain

## Abstract

**Introduction:**

The rapid evolution of SARS-CoV-2 has posed a challenge to long-lasting immunity against the novel virus. Apart from neutralizing function, binding antibodies induced by vaccination or infection play an important role in containing the infection.

**Methods:**

To determine the proportion of wild-type (WT)–generated antibodies recognizant of more recent variants, plasma samples from either SARS-CoV-2 WT-infected (n = 336) or double-mRNA (Comirnaty)–vaccinated individuals (n = 354, age and sex matched to the convalescent group) were analyzed for binding antibody capacity against the S1 protein of the BA.1 omicron variant.

**Results:**

Overall, 38.59% (95% CI, 37.01– 40.20) of WT-generated antibodies recognized Omicron BA.1 S1 protein [28.83% (95% CI, 26.73–30.91) after infection and 43.46% (95% CI, 41.61–45.31) after vaccination; p < 0.001]. Although the proportion of WT-generated binding and neutralizing antibodies also binding to BA.1 is substantially reduced, the avidity of the remaining antibodies against the Omicron variant was non-inferior to that of the ancestral virus: Omicron: 39.7% (95% CI: 38.1–41.3) as compared to the avidity to WT: 27.0% (95% CI, 25.5–28.4), respectively (p < 0.001). Furthermore, we noticed a modestly yet statistically significant higher avidity toward the Omicron epitopes among the vaccinated group (42.2%; 95% CI, 40.51–43.94) as compared to the convalescent counterparts (36.4%; 95% CI, 33.42–38.76) (p = 0.003), even after adjusting for antibody concentration.

**Discussion:**

Our results suggest that an aspect of functional immunity against the novel strain was considerably retained after WT contact, speculatively counteracting the impact of immune evasion toward neutralization of the strain. Higher antibody levels and cross-binding capacity among vaccinated individuals suggest an advantage of repeated exposure in generating robust immunity.

## 1 Introduction

Since the emergence of the SARS-CoV-2 pandemic and the subsequent introduction of vaccines, the question of individual immunity against the novel virus has been a central part of the discussion of mitigating the disease worldwide. Owing to genetic instability common to most RNA viruses, SARS-CoV-2 has been mutating at a constant pace since its emergence in December 2019.

Its high mutagenicity resulting in multiple emerging variants of concern (VOCs) has posed a challenge to vaccine- and/or infection-induced long-term immunity. In particular, the appearance of the Omicron strain represented a turn in pre-established SARS-CoV-2 immune defense, as it presented as the most antigenically distinct variant since the emergence of VOCs with the majority of the mutations affecting the S1 subdomain of the spike protein, enabling various degrees of immune escape and a potential reduction in long term protection against reinfection (1–9).

Although a number of previous studies clearly showed a greatly reduced neutralizing capacity of antibodies induced by earlier variants or vaccination (1, 3–5, 9–13), data on avidity of persisting antibodies are scarce. Antibody avidity refers to the binding strength to the target antigen and is a crucial feature of functionality. Although both binding and neutralizing antibodies have been described to wane over time (13–19), avidity has been shown to increase with the duration after contact through B cell maturation at germinal centers (14–16), thereby possibly offsetting the waning effect to an extent.

With this study, we seek to characterize concentration and binding affinity of wild-type (WT)–induced antibodies against the Omicron subvariant BA.1 (OM). For this purpose, we used plasma samples obtained from WT convalescents with no history of vaccination and age/sex-matched vaccinees who received two doses of Comirnaty (BioNTech/Pfitzer) and had no previous history of infection. Blood samples were obtained approximately 7–8 months after antigen contact in all participants adding to the comparability of the convalescent and vaccinated groups.

## 2 Methods

### 2.1 Study population and sample collection

#### 2.1.1 Convalescent group

The post-infection cohort is composed of the seropositive adult participants of the Ischgl-2 seroprevalence study (20). Seropositivity in this cohort was determined during the so-called “first infection wave” in March/April 2020. Blood samples for this study were taken in November 2020 (7–8 months after pathogen contact). Infection was ascertained by anti-S and anti-N seropositivity, and none of the included participants reported a second infection at the time of sampling.

#### 2.1.2 Vaccination group

This cohort consists of a subset of the ShieldVacc-2 study participants (21, 22), age- and sex-matched to the post-infection cohort. Participants in this group had received their second dose of Comirnaty (BioNTech/Pfitzer) SARS-CoV-2 mRNA vaccine in March/April 2021. Blood samples were taken in November 2021 (7–8 months after antigen contact). Only participants vaccinated with two doses of Comirnaty given 4 weeks apart and no history of SARS-CoV-2 infection at the time of sampling were included in the present analysis. In addition to questionnaire-based information, we excluded previous infection using an anti-N antibody assay.

### 2.2 Serological testing

#### 2.2.1 Anti–spike-1 IgG

##### 2.2.1.1 Wild type

Anti–SARS-CoV-2-QuantiVac-ELISA (IgG) (EUROIMMUN, Lübeck, Germany, CE-marked) was used to measure anti-S immunoglobulin G (IgG) antibodies targeting WT S1 protein (WT anti-S1) according to the manufacturer in a fully automated manner (Euroimmun Analyzer I, Lübeck Germany). In brief, plasma controls, calibrators, and diluted samples were incubated in wells precoated with S1 domain of SARS-CoV-2. Peroxidase-conjugated anti-human IgGs were used for the detection of specific antibodies that remain bound after three wash steps. The concentration [relative units per milliliter (RU/mL)] was calculated using six standards of known concentration provided by the manufacturer. A sample is considered to be positive if the concentration exceeds 11 RU/mL.

##### 2.2.1.2 Omicron (BA.1)

Anti–SARS-CoV-2-Omikron-ELISA (IgG) (EUROIMMUN, Lübeck, Germany, CE-marked) was used to measure in the same manner as the WT anti-S IgG antibody test above with the only exception that the plates are coated with Omicron BA.1 S1 subunit (OM). Samples are considered positive above the cutoff at 11 RU/mL.

#### 2.2.2 Avidity testing

##### 2.2.2.1 Wild type

Plasma was diluted 1:101 in a sample buffer and transferred into two microtiter wells each, which were precoated with WT S1 protein. One well was incubated with urea at 5.5 M concentrations for 10 min, whereas the other was left untreated (PBS treated as control). Antibody avidity was calculated as ratio of urea treated and untreated sample optical density in percentages. Samples exceeding the linear range limit of 2.5 were retested in appropriate dilution of 401. The test was conducted in a fully automated manner on Immunomat (Virion/Serion, Würzburg Germany).

##### 2.2.2.2 Omicron (BA.1)

Avidity testing for Omicron was conducted in the same manner as the WT avidity testing, except with wells precoated with OM BA.1 S1 protein.

##### 2.2.2.3 Ascertaining the validity of the method

To present accurately comparable avidity results, we conducted internal validation of our chosen urea assay, using 17 paired samples of convalescent subjects taken 6 months apart (**Supplementary Figure 1**). Test runs both for WT and for OM avidity at 5.5 M urea concentration showed that there was a significant increase in urea resistant fraction of antibodies over time (baseline to follow-up sample). In addition, a test run using sample triplets (**Supplementary Table 1**) confirmed on the intra-assay validity of the method chosen as the mean coefficient of variation lay below 10% [mean (median): 7.6% (8.7%)].

#### 2.2.3 Neutralization assay

Pseudovirus neutralization assays based on a replication defective vesicular stomatitis virus (VSV) were performed as earlier described (23). Briefly, VSVΔG virus expressing Green Florescent Protein (GFP) as marker gene was pseudotyped with a C-terminally truncated spike of either Wuhan (WT) or BA.1 Omicron variant. Heat-inactivated plasma samples were serially four-fold diluted starting at a 1:16 dilution and mixed with virus, to result in ~100–200 spots in control wells without serum. After 1 h of pre-incubation, plasma/virus mixes were used to infect sub-confluent 293T cells overexpressing Angiontensin converting enzyme 2 (ACE2), which were seeded one day in 96-well plates. Approximately 16 h after infection plates were analyzed in an ImmunoSpot S6 Ultra-V reader and FluoroSpot software (CTL Europe GmbH, Bonn, Germany), and infected cells were counted. Continuous 50% neutralization titers were calculated using a non-linear regression (GraphPad Prism 9.0.1, GraphPad Software, Inc., La Jolla, CA, USA). Titers > 16 were considered positive. Titers < 1 were set to 1.

### 2.3 Ethical statement

The study was approved by the Ethics Committee of the Medical University of Innsbruck (EC numbers 1330/2020 and 1168/2021), and the participants gave their informed consent to be included in the research.

### 2.4 Statistical analysis

Descriptive statistics were used for characterizing demographic data and antibody levels {mean [standard deviation (SD)] and geometric mean (GM) [95% confidence interval (CI)]}. We estimated Spearman’s correlation between WT- and OM-binding anti-S1 antibody levels as well as binding affinities to WT versus OM S1 protein. Non-parametric paired Wilcoxon test or non-parametric unpaired Kruskal–Wallis test was used to assess significant differences between immune parameters and predicting independent variables. We used multivariable linear regression model to characterize antibody concentrations as well as avidity values across multiple independent variables like age, sex, number of days after contact, and mode of antibody acquisition. Using hierarchical linear regression approach, we determined potential independent roles of variables, in particular mode of antibody acquisition, in predicting the outcome after accounting for the rest of the covariables. Statistical analysis was performed using SPSS (Version 25.0. IBM Corp., Armonk, NY, USA) and Graphpad Prism 9.3.0 (Graphpad Software Inc., La Jolla, CA, USA). A p-value of <0.05 is defined as statistically significant.

## 3 Results

### 3.1 Baseline information

Samples consisted of a total of 690 [n = 354 double mRNA–vaccinated (Comirnaty, BioNTech/Pfitzer), age- and sex-matched to n = 336 WT-infected] individuals. Groups did not differ significantly in terms of age and sex; median age of participants was 46 for either group, and both groups had a slightly higher proportion of male participants (51.8% in the infected cohort and 52.8% in the vaccinated cohort). Mean number of days between potential pathogen/vaccine contacts before sampling was 222.65 (SD, 10.68) and 219.63 (SD, 1.6), respectively. Median anti-S1 IgG antibody levels were significantly higher among the vaccinated group (**Table 1**).

**Table 1:** Baseline characteristics and anti-S1 IgG antibody levels.

	Convalescent group	Vaccination group	P-value
Total n = 690 (%)	336 (48.7)	354 (51.3%)	
**Age** Range Median Mean (SD)	18–91 46 45.08 (15.23)	18–91 46 45.46 (15.33)	0.819
**Sex (%)** Male Female	174 (51.8) 162 (48.2)	187 (52.8) 167 (47.2)	*0.785*
**Days since contact (infection/second vaccination)** Range Median Mean (SD)	211-243* 224* 222.65 (10.68)*	214-240 220 219.63 (1.60)	***<0.001***
**Anti-S WT S1** Median Mean (SD) Geometric mean (95% CI) Positive (≥11 RU/mL), n (%)	11.85 23.90 (39.87) 13.11 (11.83–14.64) 173 (51.49)	56.70 101.90 (174.64) 60.88 (55.49–67.16) 347 (98.02)	***<0.001***
**Anti-S OM S1** Median Mean (SD) Geometric mean (95% CI) Positive (≥11 RU/mL), n (%)	3.51 7.21 (14.37) 4.06 (3.70–4.47) 51 (15.18)	23.03 53.44 (147.57) 24.67 (22.42–27.63) 291 (82.20)	***<0.001***

*Data available for 60 participants; calculated with dates of PCR test if provided by participants; others were confirmed seropositive and had to have been infected at approximately the same time, because the region was hit by a cluster wave. Very first cases in Austria did not emerge considerably earlier and data were assessed in April 2020. Statistical significance (bold) was assumed at a p-value ≤0.05. SD, standard deviation; CI, confidence interval; RU, relative units; WT, wild type; OM, Omicron; S1, Spike subdomain 1.

### 3.2 Overall cross binding capacity

**Figure 1:**
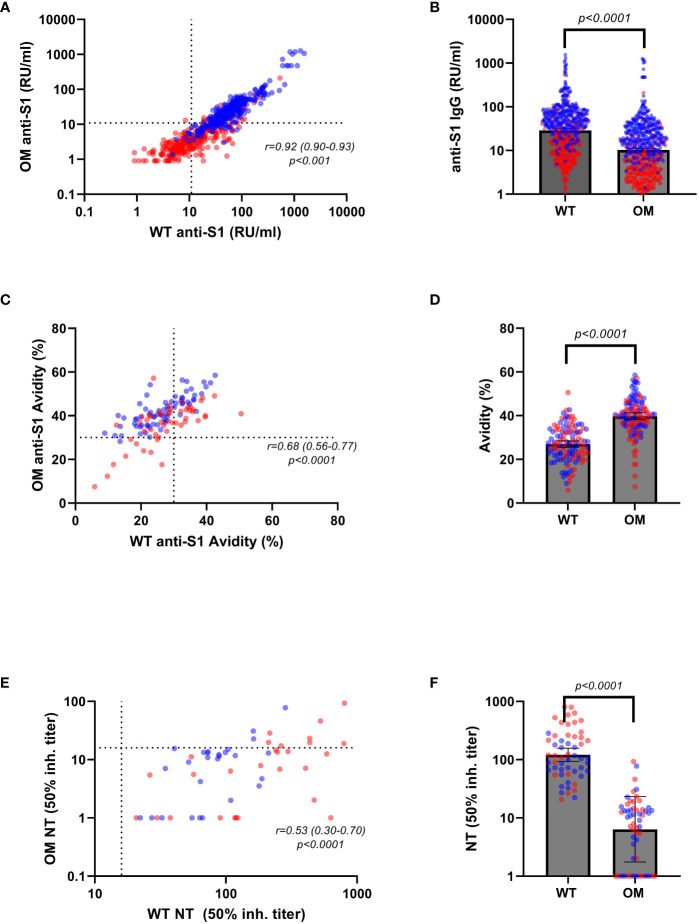
Anti-S1 IgG antibody levels, avidity indices, and neutralizing titers by variants. Serum levels of Omicron (OM) and wild-type (WT) antibodies (RU/mL) in convalescent (red) and vaccinated (blue) individuals correlated **(A)** and compared **(B)** by variants. WT and OM anti-S1 IgG avidity (%) in convalescent (red) and vaccinated (blue) individuals correlated **(C)** and compared **(D)** by variants. WT and OM neutralizing antibody titers in convalescent (red) and vaccinated (blue) correlated **(E)** and compared **(F)** by variants. Spearman’s correlation coefficients were calculated to assess correlations between binding antibody levels and avidity indices toward different variants **(A, C, E)**. Absolute differences in antibody concentration and avidity index in respect to variants were calculated using non-parametric Wilcoxon test **(B, D, F)**. Statistical significance was assumed at a p-value ≤ 0.05. RU, relative units.

Approximately 7–8 months after antigen contact, 75.36% (n = 520/690) of individuals tested positive for anti-WT S1 antibodies and 49.6% (n = 342/690) subjects tested positive for OM specific anti-S1 antibodies (**Table 1**, **Figures 1A, B**). In individuals, who tested positive for anti-WT S1 antibodies, we found that a mean 38.59% (CI, 37.01–40.20) of WT-elicited antibodies—either through vaccination or infection—also bind to OM S1 epitopes with significant differences between modes of acquisition and age group above/below 50 years. Neither sex nor having symptomatic infection/experiencing systemic reactivity after vaccination predicted binding capacity (**Table 2**).

**Table 2:** Cross-binding capacity of wild-type generated antibodies toward OM S1 epitopes among WT S1–positive samples.

	n	Mean % OM-binding IgG (95% CI)*	P-value
Overall (WT IgG positive)	520	38.59 (37.01–40.20)	
Sex			
Male Female	280 240	37.63 (35.44–39.84) 39.71 (37.55–41.82)	*0.183*
Mode of antibody acquisition			
WT infection mRNA vaccine (double dose)	173 347	28.83 (26.73–30.91) 43.46 (41.61–45.31)	***<0.001***
Age			
<50 ≥50	293 227	39.93 (37.98–41.75 36.86 (34.38–39.35)	***0.049***
Any symptoms (convalescent)**	173		
Yes No	155 18	28.92 (26.66–31.16) 28.05 (20.44–36.25)	*0.812*
Any systemic reactivity (vaccinated)***	347		*0.132*
Yes No	183 164	44.76 (42.29–46.98) 42.00 (39.41–44.87)	

*Refers to the proportion of WT-generated antibodies that also bind to OM S1 protein.

**Reported symptoms include fever, cough, breathing difficulties, sore throat, loss of smell or taste, and Gastrointestinal (GI) symptoms.

***Reported symptoms include fever, headache, chills, night sweats, vomiting, myalgia, and fatigue.

Statistical significance (bold) was assumed at a p-value ≤ 0.05. WT, wild type; OM, Omicron; S1, Spike subdomain 1; CI, confidence interval.

### 3.3 Binding affinity

For the analysis of binding affinity, we included all the samples that had tested positive for anti-OM IgG (n = 51) from the convalescent group (**Table 1**), as well as 69 samples from the vaccinated group. Selected samples from the vaccinated cohort were OM IgG antibody concentration-matched to the convalescent samples to prevent any influence of antibody level on avidity determination and guarantee comparability of the avidity maturation progress, because the antibody concentrations have been shown to affect avidity outcomes (24).

Contrary to our findings of higher antibody concentrations toward the WT S1 protein [GM (95% CI): 28.83 (26.36–31.84) vs. 10.24 (9.31–11.33); p ≤ 0.001], mean binding affinity was shown to be greater toward the OM epitopes than to the WT epitopes [mean (95% CI): 39.70 (38.04–41.23) vs. 26.97 (25.46–28.34); p < 0.001] (**Table 3**, **Figures 1C, D**). This finding remained significant in a multivariable regression analysis that adjusted for age, sex, and days after contact (**Supplementary Tables 2, 3**).

**Table 3:** Baseline characteristics and avidity of subcohort.

	Convalescent group	Vaccination group	P-value
Total n = 120 (%)	51 (42.5)	69 57.5)	
**Age** Range Mean (SD) Median	18–78 50.57 (15.58) 55	18–70 40.20 (15.00) 37	***<0.001***
**Sex (%)** Male Female	33 (64.7) 18 (35.3)	54 (78.3) 33 (21.7)	*0.147*
**Days since contact (infection/second vaccination)** Range Median Mean (SD)	211–239* 225* 222.67 (9.90)*	217–228 219 219.45 (1.42)	***0.011***
**Anti-S WT S1** Median Mean (SD) Geometric Mean	50.24 75.30 (79.23) 56.43 (46.56–69.51)	51.57 67.94 (49.71) 55.50 (47.47–64.11)	*0.534*
**Anti-S OM S1** Median Mean (SD) Geometric Mean (95% CI)	16.86 26.81 (29.85) 20.94 (17.89–24.95)	19.32 26.70 (19.26) 20.99 (17.48–24.87)	*0.980*
**Avidity WT** Median Mean (SD) Geometric Mean (95% CI)	27.89 27.62 (8.78) 25.93 (22.88–28.78)	26.60 26.49 (7.67) 25.27 (23.41–27.18)	*0.455*
**Avidity OM** Median Mean (SD) Geometric Mean	38.13 36.35 (9.47) 34.60 (31.04–37.76)	40.65 42.19 (7.17) 41.59 (40.04–43.29)	***0.003***

*Data available for nine participants; calculated with dates of PCR test if provided by participants; others were confirmed seropositive and had to have been infected at approximately the same time, because the region was hit by a cluster wave. Very first cases in Austria did not emerge considerably earlier and data were assessed in April 2020.

Statistical significance (bold) was assumed at a p-value ≤ 0.05. SD, standard deviation; CI, confidence interval; RU, relative units; WT, wild type; OM, Omicron; S1, Spike subdomain 1.

### 3.4 Neutralization testing

For a subcohort of 55 (randomly selected from the avidity dataset) samples [n = 28 (50.9%) convalescent], we evaluated neutralizing capacity toward WT and OM BA.1 strain (**Figures 1E, F**). Expectedly, we found that neutralization capacity from WT to OM was greatly reduced, with only 11 (20%) samples testing positive (cutoff ≥ 16) for OM neutralization titer, as compared to 100% (n=55) of samples that showed positive neutralization titers for WT epitope. When compared by Omicron neutralization status, positive samples showed statistically significantly higher binding antibody titers against WT (GM 140.81 vs. 66.96; p < 0.001) and Omicron (GM 44.94 vs. 27.48; p = 0.004), as well as higher avidity levels toward both WT (mean 34.14 vs. 26.55; p = 0.006) and OM (mean 43.17 vs. 39.34; p = 0.202) (**Table 4**).

**Table 4:** Characterizing binding antibody concentration and avidity for wild-type and Omicron variant across neutralization status.

		Wild-type			Omicron		
	n*	NT titer (GM, 95% CI)	Anti-S1 IgG (GM, 95% CI)	Avidity (mean, 95% CI)	NT titer (GM, 95% CI)	Anti-S1 IgG (GM, 95% CI)	Avidity (mean, 95% CI)
Overall (WT NT positive)	55	121.26 (94.03–156.20)	77.69 (64.26–93.25)	28.07 (25.74–30.25)	6.38 (4.50–8.69)	30.32 (25.53–36.13)	40.11 (37.61–42.50)
**OM NT** **Positive (>16)**	11	334.26 (243.72–475.21)	140.81 (100.59–202.57)	34.14 (29.14–38.97)	30.28 (22.34–43.91)	44.94 (26.73–72.35)	43.17 (38.01–48.55)
**OM NT** **Negative (<16)**	44	94.11 (73.16–122.19)	66.96 (55.22–81.19)	26.55 (24.35–29.00)	4.32 (3.04–5.85)	27.48 (23.02–32.59)	39.34 (36.87–41.95)
P-value**		***<0.001***	***<0.001***	***0.006***		***0.004***	*0.202*

All samples tested for neutralizating function for both WT and OM BA.1 variants are shown here with their respective binding antibody levels and avidity. All samples showed neutralizing activity against WT epitopes, whereas only 11 samples tested positive for Omicron neutralization.

*n = 55 is based on randomly selected samples from the avidity analysis sub-group.

**P-values for differences between the OM NT–positive and OM NT–negative groups. Statistical significance (bold) was assumed at a p-value ≤ 0.05.

Neutralization titer (NT) refers to the highest dilution resulting in 50% reduction in signal reduction.

WT, wild type; OM, Omicron; GM, geometric mean; CI, confidence interval; S1, spike subdomain 1.

### 3.5 Analysis across mode of antibody acquisition (convalescent versus vaccinated)

**Figure 2:**
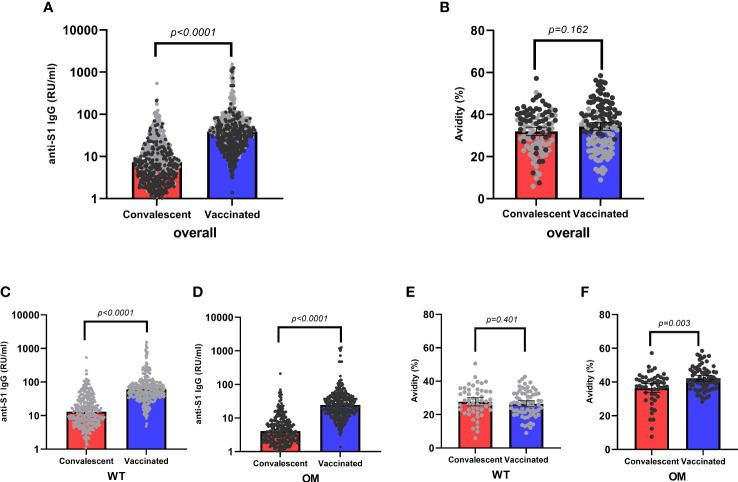
Anti-S1 IgG antibody levels and avidity indices by mode of acquisition. Overall antibody levels (RU/mL) **(A)** and avidity (%) **(B)** toward wild-type (WT, gray) and Omicron epitopes (OM, black) by mode of acquisition (convalescent, red; vaccinated, blue). WT **(C)** antibody levels and OM **(D)** antibodies by mode of acquisition. Avidity indices of WT S1 IgG **(E)** and avidity indices of OM S1 IgG **(F)** by mode of acquisition. Kruskal–Wallis test was used to assess absolute differences in antibody concentration and avidity toward different variants in respect to different modes of acquisition. Statistical significance was assumed at a p-value ≤ 0.05. RU, relative units.

Of 520 WT positive tested samples, 173 (33.3%) were convalescent samples, whereas 347 (66.7%) were vaccinated. When tested for OM BA.1, of the 342 positive samples, 51 (14.9%) were from convalescent and 291 (85.1%) from vaccinated individuals (**Table 1**). Antibody concentration for both WT and OM subtypes were significantly higher among vaccinated subjects as compared to the convalescent group (**Table 1**, **Figures 2A, C, D**) . Likewise, the proportion of WT-generated antibodies also binding to OM S1 was higher among vaccinated subjects (**Table 2**).

Avidity, on the other hand, was only modestly higher among the vaccinated and only statistically significant for avidity to OM S1 (**Table 3**, **Figures 2B, E, F**). After accounting for differences in age, sex, and antibody concentration in a hierarchical regression analysis model, vaccination remained an independent predictor of higher avidity to the OM S1 (**Supplementary Table 4**).

## 4 Discussion

Because the Omicron variant carries a decent number of mutations on the spike protein with respect to the original strain, it is expected that not the entirety of binding antibodies generated after WT contact would recognize the S1 domain of the Omicron variant. Our results showing lower binding and neutralizing antibody titers toward the OM epitopes in our main analysis reflect these findings and extend on the work of Carreno et al. (12), showing a reduced binding capacity from WT infection–generated antibodies toward Omicron receptor binding domain (RBD).

Although lower titers toward the mutated antigen were expected, what did come as a surprise was the fact that antibody avidity to the Omicron variant was found to be not only non-inferior to WT avidity. In fact, binding affinity was indeed slightly higher toward OM antigen across all tested cohorts Whether or not any mutation-associated structural change of epitopes contributes to this observation cannot be determined on the basis of the data obtained in this study and may warrant further studies scrutinizing the molecular aspects of this interaction. However, considering the fact that the contact of these cohorts was limited to WT antigen only, this finding is worthy of note, especially considering the findings of other studies (12, 25) showing that pre-existing non-neutralizing antibodies and antibody avidity seem to have a protective role against severe disease course and may, among others, also play a role in the attenuated severity of the Omicron era (26, 27). Similar data involving all other relevant VOCs may shed light on this presumption if the higher binding affinity correlates across variants.

Given that sample acquisition took place 7–8 months after antigen contact, a reduced percentage of individuals testing positive even for WT protein can be anticipated. Our results of an overall 75% of WT positive individuals fits well into research showing considerable IgG antibody waning after an initial peak within the first 3 months after antigen contact. Data on antibody dynamics have further shown initially high IgG levels (especially after vaccination) to decay rapidly, eventually plateauing into stable levels, persisting up to 1 year and beyond (19, 28–32).

### 4.1 Mode of acquisition

Structural changes following mutation may impact binding capacity and functionality, in our study resulting in a reduced yet substantial preservation of binding capacity toward the mutated variant both in the convalescent and vaccinated groups. Although a solid correlation could be shown between OM and WT anti-S (S1) antibody titers across both groups, vaccinees presented with higher titers overall. Similar results have been found by Carreno et al. (12), who found, in their study, that one-third of convalescents tested positive for antibodies against the mutated antigen and titers were more than 7.5-fold reduced as compared to the ancestral protein. In double mRNA (Comirnaty)–vaccinated individuals, reduction was 2.5-fold. Compared to our study, higher positivity rates among convalescents as found by these authors may arise from testing different epitopes as well as earlier sampling, as convalescent and double vaccinated samples were taken no more than 3 months after contact.

Antibodies generated following vaccination also seem to have slightly superior binding affinity as compared to the post-convalescent ones at the same concentrations. Considering that antibody titers impact avidity results (24), our approach of concentration-matched avidity testing allows for better comparability of the binding affinity. Because avidity maturation is a time-dependent process, differences in the time-period between antigen contact and blood sampling between the vaccinated and convalescent groups might impact results. However, with a median of 225 days after infection in the convalescent cohort and 219 days after vaccination in the vaccinated group, this difference is highly unlikely to affect outcomes. Study groups also differed in age, yet multivariable analysis revealed no confounding impact of said age differences between study cohorts on avidity results, a finding supported by a previous study from our group that showed no difference in avidity maturation across age (33). We consider higher avidity in vaccinated individuals likely to reflect a possible benefit of repeated exposure to the antigen (28, 34, 35); a perception also in line with our finding that vaccination was shown to be a predictor of avidity maturation toward the new variant, independent of antibody titer.

### 4.2 The role of binding antibodies

Although neutralizing antibodies are considered the protective class, binding antibodies have been shown to correlate with protection against SARS-CoV-2 (36–38), highlighting their importance in immune response against SARS-CoV-2. In particular, Fc effector functions like antibody-dependent cell-mediated cytotoxicity and antibody-dependent cell-mediated phagocytosis seem to play a vital role (12, 38–40). Bahnan et al. (39) found significant protective properties of non-neutralizing antibodies conferred by enhanced phagocytosis, and Bates et al. show that neutralization breadth across variants was also linked to Fc effector functions, adding to the significance of characterizing antibodies beyond neutralization function (40). Furthermore, (binding) antibody retention against mutated variants can be argued to harbor protective potential even at low concentrations, because it signifies the presence of memory B cells whose activity can be ramped up by new contact.

Our characterization of neutralizing function showed a greatly reduced neutralizing capacity from WT to Omicron variants as was expected from previous reports showing the great immune evasion potential of the Omicron strain (1, 3–5, 8–13). Because neither vaccination nor past infection could be shown to confer sterilizing immunity, serum-neutralizing activity cannot solely be regarded as the key immune function (25, 41–43). Thus, looking at other parts of immune function such as binding affinity can provide insights crucial for understanding immunity against SARS-CoV-2 and preventing (severe) disease.

### 4.3 Strengths and limitations

Although we took considerable measures to rule out prior infection in the vaccinated cohort by both assessing participants’ history of SARS-CoV-2, as well as testing for anti-N antibodies, there remains a residual risk of past infections that might affect results. Another limitation of our research may be the fact that the Enzyme-Linked Immunosorbent Assay (ELISA) assay was coated with a particular Omicron subvariant (BA.1) limiting the generalizability of our finding to other Omicron subvariants. However, antigenic mapping studies revealed Omicron subvariants to be antigenically distinct to previous VOCs (44–46), and individuals responding to the BA.1 subvariant have been shown to exhibit more comparable humoral immune response among other Omicron subvariants as opposed to more antigenically distinct pre-Omicron variants and vice versa (44, 47). This relation between Omicron subvariants is also reflected in clinical data, showing a similar disease severity among Omicron subvariants (26).

Our study focuses on humoral aspects of immunity. Because attenuated course of SARS-CoV-2 following an infection is primarily due to pathogen-specific T-cell response, it is indeed a limitation that we lack data on cellular immunity, which might have helped better characterize our finding on the role of higher antibody avidity toward the Omicron variant. We encourage further inquiry into potential confounding or a synergistic effect of these two aspects of immunity on SARS-CoV-2 disease outcome.

One major strength of this study is its large sample size and the comparability of age and sex distribution across cohorts, ruling out possible bias that may arise from biological or demographic differences. The large sample size enabled the selection of a meaningful comparison group of titer-matched samples for avidity testing across mode of antibody acquisition. This helped circumvent the genuine impact of antibody titer on avidity outcomes. A further strength of the study is that the ELISA assays used are validated according to the European standards and approved for diagnostic testing. In contrast to previous studies presenting data collected at various points in time after contact, samples in our study were taken uniformly at the approximately same time after antigen contact, providing better grounds for genuine characterization and comparison.

## 5 Conclusion

Although there is significant antigenic drift between the SARS-CoV-2 WT and Omicron BA.1 variants, our study showed that a non-negligible proportion of binding antibodies elicited after WT contact (both WT infection and double mRNA vaccination) was able to recognize the Omicron BA.1 variant, with a non-inferior binding affinity to the mutated epitopes 7–8 months after contact. Considering the importance of highly functional binding antibodies in combatting viral infections, this can be regarded as a relevant part of immunity. However, our results may not warrant a presumption of attenuated severity of the Omicron era of the SARS-CoV-2 pandemic without similar data examining other VOCs with distinct disease course.

## Data availability statement

The raw data supporting the conclusions of this article will be made available by the authors, without undue reservation.

## Ethics statement

The studies involving human participants were reviewed and approved by Ethikkommission der Medizinischen Universität Innsbruck. The patients/participants provided their written informed consent to participate in this study.

## Author contributions

Conceptualization: WB. Data acquisition: WB, TH, DB, and BF. Laboratory: WB, VF, MH, AR, and MS. Data analysis and Interpretation: WB and TH. Writing—original draft: WB and TH. Writing—revision and editing: WB, TH, BF, JK, DL, HB, DB, AR, VF, MH, MS, and RW. All authors contributed to the article and approved the submitted version.

## Supplementary material

Supplementary Figure 1 Course of anti-S IgG avidity over a period of 6 months using samples from wild-type convalescent individuals. A &B: rise in IgG antibody avidity towards wild-type S1. C & D: rise in IgG antibody avidity towards Omicron BA.1. S1. i1 samples were taken approximately 4-6 weeks post pathogen contact and i2 samples exactly 6 months after the baseline (i1) sampling. WTAV= wild-type avidity, OMAV=Omicron (BA1) avidity i1=Ischgl 1, i2= Ischgl 2, S1= Spike protein 1.
