# Supplementary figures and images for: Retained avidity despite reduced cross-binding and cross-neutralizing antibody levels to Omicron after SARS-COV-2 wild-type infection or mRNA double vaccination
Source: Front Immunol. 2023 Jul 21;14:1196988. doi: 10.3389/fimmu.2023.1196988 (PMC10401431; doi:10.3389/fimmu.2023.1196988)

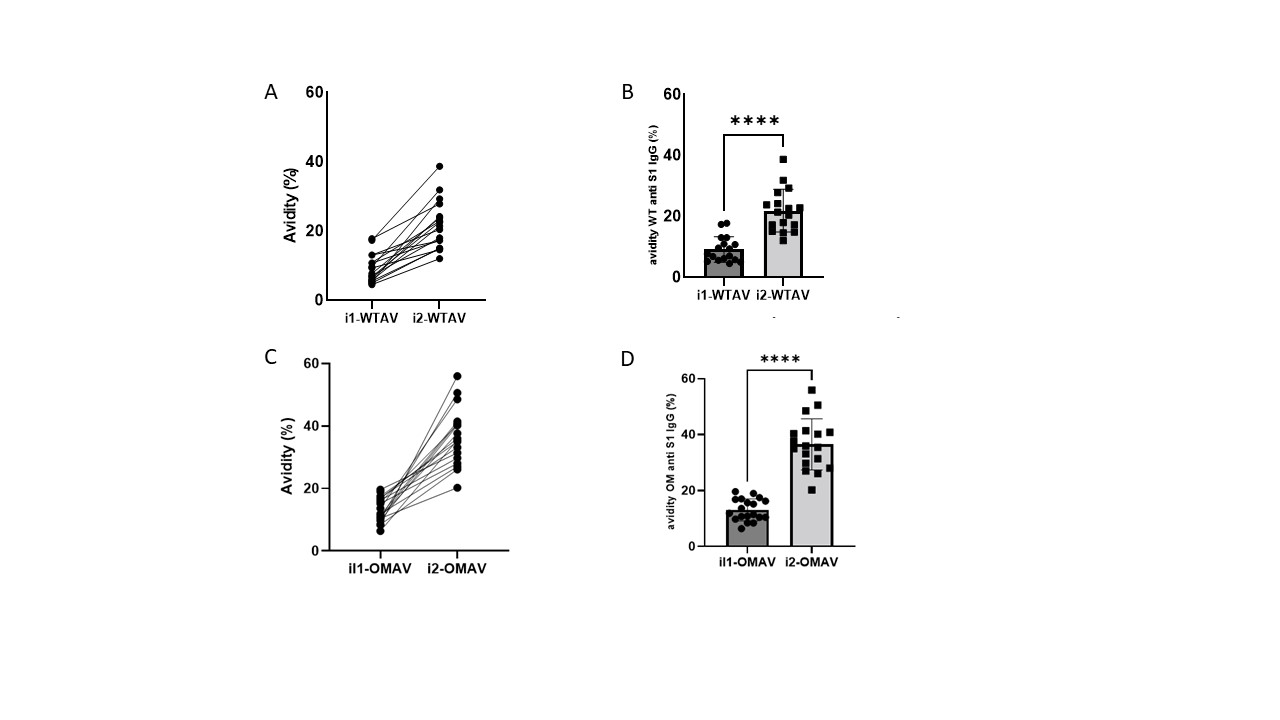

Supplement: Supplementary Figure 1 — Course of anti-S IgG avidity over a period of 6 months using samples from wild-type convalescent individuals. A &B: rise in IgG antibody avidity towards wild-type S1. C & D: rise in IgG antibody avidity towards Omicron BA.1. S1. i1 samples were taken approximately 4-6 weeks post pathogen contact and i2 samples exactly 6 months after the baseline (i1) sampling. WTAV= wild-type avidity, OMAV=Omicron (BA1) avidity i1=Ischgl 1, i2= Ischgl 2, S1= Spike protein 1. [file Image_1.jpeg]
