# Supplementary material for: Retained avidity despite reduced cross-binding and cross-neutralizing antibody levels to Omicron after SARS-COV-2 wild-type infection or mRNA double vaccination
Source: Front Immunol. 2023 Jul 21;14:1196988. doi: 10.3389/fimmu.2023.1196988 (PMC10401431; doi:10.3389/fimmu.2023.1196988)
Supplement: Supplementary file 2 [file Table_1.docx]

**Supplementary table 1: Repeatability of avidity using sample triplets***

| Sample ID** | OD PBS | OD Urea^§^ | Avidity (%) | Mean (%) | SD | CV % |
| --- | --- | --- | --- | --- | --- | --- |
| 86070242 | 0,321 | 0,050 | 15,58 |  |  |  |
| 86070242a | 0,292 | 0,057 | 19,52 |  |  |  |
| 86070242b | 0,258 | 0,046 | 17,83 | 17,64 | 1,98 | 11,10 |
| 86076949 | 0,298 | 0,051 | 17,11 |  |  |  |
| 86076949a | 0,284 | 0,044 | 15,49 |  |  |  |
| 86076949b | 0,278 | 0,041 | 14,75 | 15,79 | 1,21 | 8,20 |
| 86094318 | 0,304 | 0,054 | 17,76 |  |  |  |
| 86094318a | 0,245 | 0,034 | 13,88 |  |  |  |
| 86094318b | 0,237 | 0,037 | 15,61 | 15,75 | 1,95 | 12,47 |
| 86095481 | 0,436 | 0,083 | 19,04 |  |  |  |
| 86095481a | 0,397 | 0,077 | 19,40 |  |  |  |
| 86095481b | 0,582 | 0,112 | 19,24 | 19,23 | 0,18 | 0,94 |
| 86097180 | 0,616 | 0,184 | 29,87 |  |  |  |
| 86097180a | 0,581 | 0,194 | 33,39 |  |  |  |
| 86097180b | 0,556 | 0,168 | 30,22 | 31,16 | 1,94 | 6,42 |
| 86097259 | 0,549 | 0,126 | 22,95 |  |  |  |
| 86097259a | 0,483 | 0,099 | 20,50 |  |  |  |
| 86097259b | 0,474 | 0,090 | 18,99 | 20,81 | 2,00 | 10,54 |
| 86101353 | 0,898 | 0,110 | 12,25 |  |  |  |
| 86101353a | 1,020 | 0,121 | 11,86 |  |  |  |
| 86101353b | 0,789 | 0,103 | 13,05 | 12,39 | 0,61 | 4,66 |
| 86102707 | 0,798 | 0,105 | 13,16 |  |  |  |
| 86102707a | 0,744 | 0,116 | 15,59 |  |  |  |
| 86102707b | 0,728 | 0,098 | 13,46 | 14,07 | 1,33 | 9,85 |
| 86103986 | 0,437 | 0,141 | 32,27 |  |  |  |
| 86103986a | 0,471 | 0,133 | 28,24 |  |  |  |
| 86103986b | 0,465 | 0,128 | 27,53 | 29,34 | 2,56 | 9,28 |
| 86107609 | 0,781 | 0,258 | 33,03 |  |  |  |
| 86107609a | 0,750 | 0,238 | 31,73 |  |  |  |
| 86107609b | 0,682 | 0,223 | 32,70 | 32,49 | 0,68 | 2,07 |

*avidity using wild-type ELISA plates
**Sample ID, sample IDa and sample IDb represent triplicates
^§^5.5M urea
OD= optical density, SD= standard deviation, CV= coefficient of variation

| **Supplementary table 2. Multiple linear regression analysis for effect of predictor variables on avidity level** | | | | | | |
| --- | --- | --- | --- | --- | --- | --- |
|  | | Unstandardized Coefficients | | Standardized Coefficients | t | Sig. |
|  |  | B | Std. Error | Beta |  |  |
|  | (Constant) | 14,945 | 39,034 |  | ,383 | ,702 |
|  | Days_since_contact | ,066 | ,180 | ,023 | ,366 | ,715 |
|  | Female (compared to male) | 1,270 | 1,571 | ,051 | ,808 | ,420 |
|  | Convalescent (as compared to vaccinated) | -2,322 | 1,981 | -,071 | -1,172 | ,243 |
|  | Age | -,086 | ,045 | -,123 | -1,901 | ,059 |
|  | Anti S1 IgG concentration | ,011 | ,019 | ,047 | ,608 | ,544 |
|  | Omicron S1 epitope (compared to WT epitope | 15,087 | 1,449 | ,717 | 10,414 | **,000** |
| Dependent Variable: AV (avidity to S1 Protein of SARS-CoV-2) | | | | | | |

**Supplementary table 3. Multiple linear regression analysis for effect of predictor variables on avidity level**

| **Model Summary** | | | | | | | | | |
| --- | --- | --- | --- | --- | --- | --- | --- | --- | --- |
| Model | R | R Square | Adjusted R Square | Std. Error of the Estimate | Change Statistics | | | | |
|  |  |  |  |  | R Square Change | F Change | df1 | df2 | Sig. F Change |
| 1 | ,401^a^ | ,161 | ,133 | 9,82793 | ,161 | 5,753 | 5 | 150 | ,000 |
| 2 | ,717^b^ | ,514 | ,495 | 7,50160 | ,353 | 108,459 | 1 | 149 | ,000 |
| a. Predictors: (Constant), AB, mode of AB acquisition=conv, Days_since_contact, Sex=female, Age | | | | | | | | | |
| b. Predictors: (Constant), AB, mode of AB acquisition=conv, Days_since_contact, Sex=3.0, Age, Omicron S1 epitope | | | | | | | | | |

**Supplmentary table 4. Multiple linear regression analysis for independent predictive effect of mode of antibody acquisition on avidity level across epitope of interest**

| **Model Summary^a^** | | | | | | | | | |
| --- | --- | --- | --- | --- | --- | --- | --- | --- | --- |
| **OM AV** | R | R Square | Adjusted R Square | Std. Error of the Estimate | Change Statistics | | | | |
|  |  |  |  |  | R Square Change | F Change | df1 | df2 | Sig. F Change |
| 1 | ,279^b^ | ,078 | ,028 | 7,44830 | ,078 | 1,546 | 4 | 73 | ,198 |
| 2 | ,393^c^ | ,155 | **,096** | 7,18221 | ,076 | 6,509 | 1 | 72 | **,013** |
| \| **WT AV** \|  \|  \|  \|  \|  \|  \|  \|  \|  \| \| --- \| --- \| --- \| --- \| --- \| --- \| --- \| --- \| --- \| --- \| \| 1 \| ,281^b^ \| ,079 \| ,028 \| 7,59138 \| ,079 \| 1,561 \| 4 \| 73 \| ,194 \| \| 2 \| ,295^c^ \| ,087 \| ,024 \| 7,60937 \| ,008 \| ,655 \| 1 \| 72 \| ,421 \| | | | | | | | | | |
| a. Avidity epitope | | | | | | | | | |
| b. Predictors: (Constant), Sex, Days_since_contact, Age, AB | | | | | | | | | |
| c. Predictors: (Constant), Sex, Days_since_contact, Age, AB, Ischgl_Schwaz | | | | | | | | | |

Shows that for mode of acquisition was an independent predictor of variation in OM AV even after accounting for age, sex, antibody concentration and number of days post antigen contact. It has a statistically significant 10% more independent predictive effect. None of these variables showed statistically significant effect on the variance of WT-avidity.
